# Supplementary material for: Reconciling Mining with the Conservation of Cave Biodiversity: A Quantitative Baseline to Help Establish Conservation Priorities
Source: PLoS One. 2016 Dec 20;11(12):e0168348. doi: 10.1371/journal.pone.0168348 (PMC5173368; doi:10.1371/journal.pone.0168348)
Supplement: S1 Dataset — (ZIP) [file pone.0168348.s002.zip › Taxa/Serra Norte/SN_2013/N4E/Lista N4E-13.pdf]

CAVIDADE N4E-0013

Classe	Ordem	Fam/Outros	Gên/Outros	Espécie	S-U	U-S	Zon.
Annelida	Oligochaeta			jovem		4	P/A
Arachnida	Acari	(Oribatida)		sp.2		X	P
Arachnida	Acari	(Parasitengonina)		sp.2		X	A
Arachnida	Acari	Argasidae	*Ornithodoros*	sp.1		X	P
Arachnida	Acari	Ixodidae	*Amblyomma*	*cajennense*	X		
Arachnida	Acari	Mesostigmata		jovem	X		
Arachnida	Acari	Trombiculidae		sp.39		X	P/A
Arachnida	Amblypygi	Charinidae	*Charinus*	sp.1		1	A
Arachnida	Amblypygi	Phrynidae	*Heterophrynus*	*longicornis*	X		
Arachnida	Araneae	Corinnidae	*Myrmecium*	sp.1	1	1	P
Arachnida	Araneae	Ctenidae		jovem	1		
Arachnida	Araneae	Deinopidae		sp.	X		
Arachnida	Araneae	Dipluridae		jovem	1		
Arachnida	Araneae	Filistatidae		sp.1	X		
Arachnida	Araneae	Mysmenidae		jovem		X	A
Arachnida	Araneae	Nemesiidae		sp.1	X		
Arachnida	Araneae	Nesticidae		jovem		X	A
Arachnida	Araneae	Ochyroceratidae	*Ochyrocera*	sp.1	X	X	P/A
Arachnida	Araneae	Ochyroceratidae	*Speocera*	sp.1	X	X	P/A
Arachnida	Araneae	Oonopidae	*Orchestina*	sp.1	X	X	P
Arachnida	Araneae	Pholcidae	*Mesabolivar*	sp.1	X	X	A
Arachnida	Araneae	Salticidae		sp.	X		
Arachnida	Araneae	Scytodidae	*Scytodes*	sp.	X		
Arachnida	Araneae	Theridiidae		sp.	X		
Arachnida	Araneae	Theridiosomatidae	*Plato*	sp.1	X	X	P/A
Arachnida	Opiliones	Escadabiidae		sp.2	X	X	P/A
Arachnida	Opiliones	Gonyleptidae	Pachylinae	sp.	X		
Arachnida	Palpigradi	Eukoeneniidae	*Allokoenenia*	sp.1		X	P/A
Arachnida	Pseudoscorpiones	Chernetidae	*Spelaeochernes*	sp.1	X	X	P
Arachnida	Pseudoscorpiones	Olpiidae		sp.	X		
Chilopoda	Geophilomorpha			sp.	X		
Chilopoda	Scolopendromorpha	Scolopocryptopidae		sp.4		1	P
Chilopoda	Scutigeromorpha	Pselliodidae	*Pselliodes*	sp.2		1	
Chilopoda	Scutigeromorpha	Pselliodidae	*Sphendononema*	*guildingii*	X		
Diplopoda	Polydesmida	Chelodesmidae		jovem	X	1	P
Diplopoda	Polydesmida	Fuhrmannodesmidae		jovem		X	P
Diplopoda	Polydesmida	Pyrgodesmidae		sp.1	X	X	P/A
Diplopoda	Spirostreptida	Pseudonannolenidae	*Pseudonannolene*	sp.1	X		
Entognatha	Collembola	Entomobryidae		sp.4		X	P
Entognatha	Collembola	Isotomidae		sp.1		X	P
Entognatha	Collembola	Paronellidae		sp.4		X	P
Entognatha	Collembola	Sminthuroidea		sp.2	X	X	P/A
Entognatha	Diplura	Campodeidae		sp.1	X	X	P
Entognatha	Diplura	Projapygidae		sp.1	X	X	P
Insecta	Blattodea	Blattellidae		sp.5	1	1	P
Insecta	Coleoptera	Curculionidae	Scolytinae	sp.	X	X	P/A
Insecta	Coleoptera	Lampyridae		jovem	X		
Insecta	Coleoptera	Staphylinidae		sp.33		X	A
Insecta	Coleoptera	Tenebrionidae		sp.	X		
Insecta	Diptera	Aulacigastridae		sp.		X	A
Insecta	Diptera	Chironomidae		sp.		X	A
Insecta	Diptera	Psychodidae	*Sciopemyia*	*sordellii*	X	X	P/A
Insecta	Heteroptera	Cydnidae		sp.	X	X	A
Insecta	Homoptera	Cixiidae		jovem	X	X	P
Insecta	Hymenoptera	Diapriidae		sp.1		X	P/A

Insecta	Hymenoptera	Formicidae	*Camponotus*	sp.1	X	X	A
Insecta	Hymenoptera	Formicidae	*Crematogaster*	sp.1		X	A
Insecta	Hymenoptera	Formicidae	*Cyphomyrmex*	sp.1		X	P
Insecta	Hymenoptera	Formicidae	*Odontomachus*	sp.1		X	P
Insecta	Hymenoptera	Formicidae	*Pachycondyla*	*constricta*	X		
Insecta	Hymenoptera	Formicidae	*Pachycondyla*	sp.1		X	P
Insecta	Hymenoptera	Formicidae	*Pheidole*	sp.	X		
Insecta	Isoptera	Termitidae	*Nasutitermes*	sp.	X	X	P/A
Insecta	Lepidoptera	Tineoidea		sp.	X	X	
Insecta	Orthoptera	Gryllidae	Trigonidiinae	sp.		1	P
Insecta	Orthoptera	Phalangopsidae	*Phalangopsis*	sp.	X		
Insecta	Psocoptera			jovem	X	X	P
Insecta	Thysanura	Ateluridae	Atelurinae	sp.	X		
Gastropoda	Pulmonata	Subulinidae	*Lamellaxis*	sp.	X		
Gastropoda	Pulmonata	Subulinidae		sp.3		X	P
Gastropoda	Pulmonata	Systrophiidae	*Happia*	sp.		X	P
Malacostraca	Isopoda	Armadillidae		sp.1	X	X	P
Malacostraca	Isopoda	Scleropactidae	*Circoniscus*	*buckupi*	X		
Nematoda				sp.1		X	P
Amphibia	Anura			sp.	X	2	E/P
Mammalia	Chiroptera	Phyllostomidae	*Carollia*	*perspicillata*		1	P/A
Mammalia	Chiroptera	Phyllostomidae	*Glossophaga*	*soricina*		1	P/A
Mammalia	Chiroptera			sp.	3		P/A
